# The complete chloroplast genomes of rare medical herb *Glycyrrhiza inflata* and its relative *G. aspera* (Fabaceae)

**DOI:** 10.1080/23802359.2019.1691067

**Published:** 2019-11-18

**Authors:** Lei Duan, Zhi-Rong Zhang, Shuang-Wen Deng, Hong-Feng Chen

**Affiliations:** a Key Laboratory of Plant Resources Conservation and Sustainable Utilization, South China Botanical Garden, Chinese Academy of Sciences, Guangzhou, China;; b Germplasm Bank of Wild Species, Kunming Institute of Botany, Chinese Academy of Sciences, Kunming, China

**Keywords:** Chloroplast genome, endangered species, *Glycyrrhiza aspera*, *Glycyrrhiza inflata*

## Abstract

*Glycyrrhiza inflata* is a threatened perennial herb with medicinal value, which restricts in NW China and Mongolia. Its ally species, *G. aspera*, is widely distributed from northern China to Turkey. The complete chloroplast genomes were sequenced using the Illumina Hiseq X-Ten platform. Each of the genomes lacks an inverted repeat (IR) region, containing 76 protein-coding genes, 30 tRNAs genes, and 4 rRNAs. The overall GC contents are both 34.3%. A phylogenetic tree based on the whole chloroplast genomes of 15 species indicated that *G. aspera* and *G. inflata* belonged to a monophyletic *Glycyrrhiza*, which was nested in IRLC group of the subfamily Papilionoideae (Leguminosae).

The liquorice species of *Glycyrrhiza inflata* Batal. and *G. aspera* Pall. (Fabaceae, the legume family) are perennial herbs, their long and strong roots were widely used as traditional medicine in China (Li and Cui 1998) to relieve cough and phlegm. *Glycyrrhiza inflata*, distributed in NW China and Mongolia (Yakovlev 2003; Bao and Larsen 2010), has been listed as a rare and endangered plant in the category of key protected wild plants in China (http://www.iplant.cn/rep/prot/Glycyrrhiza%20inflata). *Glycyrrhiza aspera* is a relative species of *G. inflata*, ranging from northern China to Turkey (Chamberlain 1970; Meng 2005). A good knowledge in genomic information of these liquorice species would contribute to the study of population genetics, diversity, medical use and the establishment of efficient protection strategy towards the endangered natural resource.

The fresh leaves of *G. inflata* and *G. aspera* were collected in Xinjiang, China, and the voucher specimens were deposited in the herbaria of South China Botanical Garden, Chinese Academy of Sciences (IBSC, collection #: *Duan 2016018*), and of Northwest A&F University (WUK, collection #: *Chang* et al. *2015143*), respectively. We extracted the total genomic DNA with CTAB approach (Doyle 1987), the genomic libraries were prepared and sequenced using the Illumina Hiseq X-Ten platform (Illumina Inc., San Diego, CA). The resultant sequences were filtered following Yao et al. (2016), the adaptor-free reads were then assembled with SPAdes 3.11 (Bankevich et al. 2012). We annotated the assembly of complete chloroplast (cp) genomes using the Dual Organellar GenoMe Annotator (DOGMA) (Wyman et al. 2004) and deposited the genomes in GenBank (accession number: MN562092 for *G. inflata*; MN562093 for *G. aspera*).

About 1.45 Gb and 1.17 Gb raw reads of *G. inflata* and *G. aspera* were obtained, respectively, with coverage of 750× and 127,826 bp in length for the former, 629× and 127,831 bp for the latter. Both of the cp genomes lacked an inverted repeat (IR) region. Each of them contained 76 protein-coding genes (CDS), 30 transfer RNA genes (tRNA), 4 ribosomal RNA genes (rRNA), within which 15 genes (*atpF*, *ndhA*, *ndhB*, *petB*, *petD*, *rpl2*, *rpl16*, *rpoC1*, *rps12*, *trnA-UGC*, *trnG-UCC*, *trnI-GAU*, *trnK-UUU*, *trnL-UAA*, and *trnV-UAC*) had one intron, one gene (*ycf3*) has two introns. Overall GC content of the whole genomes were both 34.3%.

To infer the phylogenetic relationships among these two species and their related taxa, whole cp genomes of 13 Papilionoideae species were downloaded from GenBank, which were aligned with those of *G. inflata* and *G. aspera* by applying MAFFT v.7 (Katoh and Standley 2013). Based on the alignment, a maximum likelihood (ML) tree was constructed using IQ-TREE v.1.6 (Nguyen et al. 2015) (Figure 1). The result showed that *G. aspera*, *G. inflata*, *G. glabra*, and *G. uralensis* constituted the monophyletic *Glycyrrhiza*. This genus was nested in the IR-lacking clade (IRLC), which in turn belonged to the clade of Hologalegina as suggested by a previous study (Wojciechowski et al. 2004).

**Figure 1.**
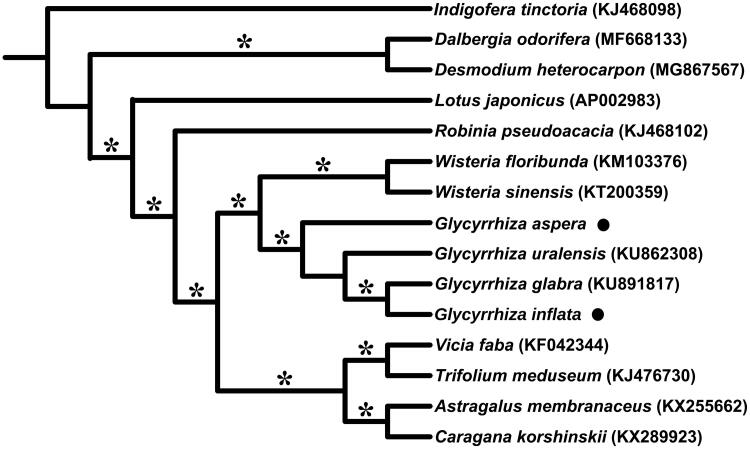
Maximum likelihood (ML) phylogenetic tree based on 15 chloroplast genomes of Fabaceae. The position of *Glycyrrhiza inflata* and *G. aspera* are indicated with black dots, respectively. The bootstrap values of 100% are shown on branches with asterisks.
